# The Pathological Findings Following Laparotomy in Hodgkin's Disease

**DOI:** 10.1038/bjc.1971.57

**Published:** 1971-09

**Authors:** Geoffrey Farrer-Brown, M. H. Bennett, C. V. Harrison, Yvonne Millett, A. M. Jelliffe

## Abstract

**Images:**


### THE PATHOLOGICAL FINDINGS FOLLOWING LAPAROTOMY IN HODGKIN'S DISEASE

GEOFFREY FARRER-BROWN, M. H. BENNETT, C. V. HARRISON, YVONNE MILLETT AND A. M. JELLIFFE

*From the Bland-Sutton Institute of Pathology and the Radiotherapy Department of The Middlesex Hospital, London; the Pathology Departments of Mount Vernon Hospital, Northwood, Middlesex and of the Royal Postgraduate Medical School, London*

Received for publication June 29, 1971

**SUMMARY.—The pathological findings in 50 patients with Hodgkin's disease following laparotomy for diagnostic purposes are described. Forty-four patients had laparotomy before treatment and within a few months of the original diagnosis, while 6 patients had delayed laparotomies. The Rye histological classification was applied to the original lymph node biopsy, the abdominal lymph node and Hodgkin's tissue in the spleen. The variation in appearance both of these tissues and of the liver biopsies is discussed.**

LAPAROTOMY as a diagnostic procedure in patients with Hodgkin's disease as advocated by Glatstein *et al.* (1969, 1970) and Jelliffe *et al.* (1970) is allowing pathologists, for the first time, to study the extent of splenic, hepatic and abdominal lymph node involvement early in the course of the disease. The variation in histological appearances in the different tissues can be compared and correlated with the original lymph node biopsy and is providing new information about the progression of the disease.

This paper reports the pathological findings in a series of 50 patients* with Hodgkin's disease at the Middlesex and Harefield Hospitals and the Royal Postgraduate Medical School, who had laparotomy for diagnostic purposes between April 1970 and May 1971. Forty-four patients had laparotomies before treatment and within a few months of the original lymph node biopsy while 6 patients had treatment before laparotomy which was performed 11 months to 12 years after biopsy.

### METHOD

All spleens were weighed, measured and cut into two or more slices depending on size, to obtain good fixation. When fixed they were examined grossly and if no obvious tumour deposits were seen the spleens were sliced as thinly as possible in order to detect single minute foci. If no abnormality was apparent the general size of the malpighian bodies was assessed and random blocks taken that included the largest malpighian bodies as these were the most likely sites of early involvement. These blocks, plus the wedge biopsy of liver and abdominal lymph node, were then routinely processed for histology.

All histological sections including the original lymph node biopsy were reviewed by three of the authors (G.F-B., M.H.B., C.V.H.).

* This series of spleens forms part of the material submitted to the National Lymphoma Investigation (Clinical Cancertherapy Cooperative Group) which commenced in February 1970.

### RESULTS

### *General findings*

The age range of the 35 males and 15 females was 11–77 years, with a median of 30 years. Laparotomy was performed within a few months of diagnosis and before treatment in 44 patients, 50% of whom were found to have invaded spleens. The details of the latter patients, with the histological type of Hodgkin's disease (Rye classification, Lukes *et al.*, 1966) of the original lymph node, the spleen weight and the involvement of the liver and abdominal lymph node biopsy are shown in Table I. Similar details of the 22 patients with uninvolved spleens are shown in Table II.

TABLE I.—*Details of 22 Pre-treatment Laparotomy Patients with Invaded Spleens*

| Rye classification of original lymph node | Case No. | Patient Initials | Sex | Age | Hospital | Spleen wt | Abd. lymph nodes | Liver involvement |
|---|---|---|---|---|---|---|---|---|
| LP | 4 | WW | M | 55 | Mx | 550 | .. | 0 |
| | 25 | AL | M | 56 | RPMS | 660 | + | + |
| | 28 | MH | F | 37 | Mx | 280 | .. | – |
| | 36 | RM | M | 52 | HH | – | + | + |
| | 37 | AS | M | 75 | HH | – | + | + |
| | 39 | SB | F | 45 | Mx | 229 | – | – |
| NS | 1 | MP | M | 16 | Mx | 385 | + | – |
| | 8 | KE | M | 15 | Mx | 254 | + | – |
| | 11 | CB | M | 59 | Mx | 175 | .. | – |
| | 12 | GC | M | 23 | Mx | 870 | + | – |
| | 13 | PJ | F | 38 | Mx | 149 | + | – |
| | 14 | CM | M | 28 | Mx | 213 | .. | – |
| | 24 | RB | M | 31 | Mx | 480 | .. | – |
| | 26 | DC | M | 35 | Mx | 206 | + | – |
| | 38 | CC | F | 24 | Mx | 394 | .. | – |
| | 43 | DE | M | 47 | RPMS | 313 | + | – |
| | 44 | HC | M | 46 | Mx | 495 | + | – |
| MC | 7 | TMG | M | 25 | Mx | 77 | .. | – |
| | 15 | JB | M | 40 | Mx | 370 | .. | – |
| | 29 | RR | M | 22 | Mx | 250 | .. | – |
| | 35 | EM | M | 58 | Mx | 505 | + | – |
| | 41 | FC | M | 77 | RPMS | 295 | + | – |

LP Lymphocytic predominant — Mx Middlesex Hospital

NS Nodular sclerotic — HH Harefield Hospital

MC Mixed cellularity — RPMS Royal Postgraduate Medical School

+ invaded — uninvolved 0 not biopsied

The original lymph node biopsy from each of these 44 patients was classified histologically according to the Rye Modification (Lukes *et al.*, 1966) of the Lukes and Butler (1966) classification. Six of 7 patients with lymphocytic predominant disease, 11 of 23 with nodular sclerotic Hodgkin's and 5 of 14 with mixed type disease had invaded spleens. There were no patients with lymphocytic depleted Hodgkin's disease in the original lymph node in this series of laparotomies.

The median weight of 20 invaded spleens was 304 g. with a range of 77–870 g. compared with a median of 192 g. and a range of 100–690 g. in the uninvolved spleens (Fig. 1). In 14 of the invaded spleens macroscopic examination showed obvious abnormal nodules, but in 2 spleens only single nodules, 2 and 3 mm.

TABLE II.—*Details of 22 Pre-treatment Laparotomy Patients with Uninvolved Spleens*

| Rye classification of original lymph node | Case No. | Patient | | | Hospital | Spleen wt | Abd. lymph nodes | Liver involved |
|---|---|---|---|---|---|---|---|---|
| | | Initials | Sex | Age | | | | |
| LP | 33 | AM | M | 21 | Mx | 195 | — | — |
| NS | 2 | EB | M | 22 | Mx | 155 | .. | — |
| | 3 | CF | F | 36 | Mx | 255 | — | — |
| | 9 | SL | F | 22 | Mx | 140 | .. | — |
| | 10 | MW | F | 11 | Mx | 100 | + | — |
| | 16 | SM | F | 24 | Mx | 255 | + | — |
| | 17 | EM | F | 20 | Mx | 205 | .. | — |
| | 18 | RP | M | 19 | Mx | 321 | .. | — |
| | 19 | EG | F | 19 | Mx | 204 | .. | — |
| | 20 | AR | M | 30 | Mx | 160 | .. | — |
| | 22 | AP | F | 29 | Mx | 105 | — | — |
| | 31 | SZ | M | 26 | Mx | 163 | .. | — |
| | 42 | WW | F | 60 | Mx | 165 | .. | — |
| MC | 5 | DB | M | 24 | Mx | 360 | .. | — |
| | 6 | MS | M | 34 | Mx | 399 | .. | — |
| | 21 | FK | M | 33 | Mx | 690 | + | — |
| | 23 | BS | M | 15 | Mx | 420 | — | — |
| | 27 | JM | M | 28 | RPMS | 180 | .. | — |
| | 30 | JC | M | 37 | Mx | 153 | .. | — |
| | 32 | KB | M | 35 | Mx | 164 | — | — |
| | 34 | AS | M | 30 | Mx | 340 | .. | — |
| | 40 | JO | M | 57 | Mx | 125 | .. | — |

LP Lymphocytic predominant
NS Nodular sclerotic
MC Mixed cellularity

Mx Middlesex Hospital
RPMS Royal Postgraduate Medical School

+ invaded — uninvolved respectively, were present (Fig. 2). An additional 3 spleens contained only 2 to 5 small foci up to 7 mm. in diameter. Three specimens with only slight prominence of the malpighian bodies and no macroscopic discrete foci proved to be positive on histological examination. All but one of these 22 patients with splenic involvement had a wedge liver biopsy at laparotomy and in three instances microscopical examination showed Hodgkin's disease. Each of these patients had lymphocytic predominant type disease in the original lymph node. Abdominal lymph nodes were biopsied in 12 patients and all but one were shown microscopically to be invaded. Of the remaining 10 cases of this group, who did not have abdominal lymph node biopsies, 3 were considered clinically at laparotomy to have enlarged invaded nodes.

Of the 22 patients with uninvolved spleens, one had lymphocytic predominant Hodgkin's, 12 nodular sclerotic and 9 mixed type disease in the original lymph node. Eight had abdominal lymph node biopsies three of which proved on histological examination to be invaded, while of the remaining 14 patients only two were found to have obviously invaded nodes at the time of laparotomy. A wedge biopsy of liver was taken from all but one patient in this group and in one case, a man of 40 with mixed type histology in the original lymph node, the liver showed an abnormal mixed cellular infiltrate extending out between parenchymal cells in the portal tracts but despite careful sectioning no diagnostic Reed-Sternberg cells were seen.

FIG. 1.—The weights of uninvolved spleens and those invaded by Hodgkin's tissue.

### EXPLANATION OF PLATES

FIG. 2.—The microscopical appearance of a solitary focus of Hodgkin's tissue in the spleen. H. and E. × 24.

FIG. 3.—Abnormal histiocytes present at the periphery of a malpighian body and in the peri-arteriolar area. H. and E. × 100.

FIG. 4.—A malpighian body diffusely infiltrated by atypical cells. H. and E. × 105.

FIG. 5.—High magnification of peri-arteriolar area to show invasion by a pleomorphic cellular tissue. H. and E. × 100.

FIG. 6.—Nodular sclerotic Hodgkin's disease in the spleen. H. and E. × 18.

FIG. 7.—An area of the same spleen as in Fig. 6 showing invasion of the malpighian bodies and peri-arteriolar areas by Hodgkin's tissue but no bands of fibrous tissue are present. H. and E. × 25.

FIG. 8.—" Hodgkin's granulomata " in a third area of the spleen illustrated in Fig. 6 and 7. H. and E. × 75.

FIG. 9.—Early involvement of a para-aortic lymph node by Hodgkin's disease with invasion confined to the para-follicular area. The reactive centre of a follicle is seen on the left of the figure. H. and E. × 75.

FIG. 10.—A small single focus of Hodgkin's tissue in the liver. H. and E. × 48.

FIG. 11.—A pleomorphic cellular infiltrate extending out amongst parenchymal cells but no Reed-Sternberg cells present. H. and E. × 75.

2

3

Farrer-Brown *et al.*

4

5

6

7

8

9

Farrer-Brown *et al.*

10

11

Farrer-Brown *et al.*

The remaining 6 patients in this study had laparotomy performed for diagnostic purposes 11 months to 12 years after the original lymph node biopsy and following radiotherapy treatment to the upper half of the body. In addition Case 48 had had a course of chemotherapy. Five of these patients had nodular sclerotic and one mixed type Hodgkin's disease in the original lymph node (Table III). The spleen weights varied from 110–735 g. but one weighing 230 g. from a patient with mixed type disease contained only a single nodule (0·8 cm. diameter) of Hodgkin's tissue. The two spleens with weights under 200 g. contained only occasional nodules up to 1 cm. in diameter although these patients had had their original lymph node biopsies 3 and 13 years previously. The three heaviest spleens all contained numerous large nodules of tumour tissue up to 8 cm. in diameter. Only one of these 6 patients had an abdominal lymph node biopsy but 4 were considered at operation to have invaded glands. In two instances the liver biopsy showed invasion by Hodgkin's tissue.

TABLE III.—*Details of Patients who had a Laparotomy 11 Months to 12 Years After Original Lymph Node Biopsy Following Radiotherapy Treatment Confined to Upper Half of Body (Case 48 also had a Course of Chemotherapy)*

All spleens invaded

| Case No. | Patients: Initials | Sex | Age | Hospital | Rye classification of original lymph node | Abd. lymph nodes | Spleen wt | Time from original lymph biopsy to splenectomy | Liver involvement |
|---|---|---|---|---|---|---|---|---|---|
| 45 | AB | M | 18 | Mx | NS | .. | 181 | 3 yrs. | — |
| 46 | LL | F | 40 | Mx | NS | + | 110 | 13 yrs. | — |
| 47 | JW | F | 27 | Mx | NS | .. | 735 | 11 mths. | + |
| 48 | VK | F | 32 | Mx | NS | .. | 388 | 3 yrs. | + |
| 49 | JT | F | 18 | Mx | NS | .. | 355 | 3 yrs. | — |
| 50 | SAE | M | 37 | Mx | MC | .. | 230 | 2 yrs. | — |

Mx Middlesex Hospital NS Nodular sclerotic MC Mixed cellularity
\+ invaded — uninvolved

*Spleens*

An attempt was made to apply the Rye histological classification to the foci of Hodgkin's tissue in the invaded spleens and comparison was made with the appearances of the original lymph node biopsy. Of the 22 patients with involved spleens removed at laparotomy before treatment 9 had lymphocytic predominant, 4 nodular sclerotic, 3 mixed cellularity and 6 lymphocytic depleted Hodgkin's disease. Table IV shows the correlation between the histological type in the

TABLE IV.—*Comparison of Histological Type of Hodgkin's Disease in the Invaded Spleens and Original Lymph Node Biopsies*

| Rye classification of original lymph node | Total No. of cases | No. with invaded spleens | Rye classification of Hodgkin's tissue in spleen: LP | NS | MC | LD |
|---|---|---|---|---|---|---|
| Lymphocytic predominant | 7 | 6 | 4 | 0 | 0 | 2 |
| Nodular sclerotic | 23 | 11 | 3 | 5 | 1 | 2 |
| Mixed cellularity | 14 | 5 | 2 | 0 | 1 | 2 |

LP, Lymphocytic predominant; NS, Nodular sclerotic; MC, Mixed cellularity; LD, Lymphocytic depleted.

spleen and the original lymph node. 45% had the same histological type of Hodgkin's disease in the spleen as in the original lymph node while in 27% a lymphocytic depleted appearance with diffuse fibrosis was present in the spleen although the lymph node histology was lymphocytic predominant, nodular sclerotic or mixed in type. In contrast 5 of the 16 patients with nodular sclerotic or mixed lymph node histology showed a lymphocytic predominant picture in the spleen.

Microscopical examination of the three spleens with no obvious gross abnormality except slightly prominent and whiter than normal malpighian bodies showed involvement of the lymphoid tissue by Hodgkin's disease of the lymphocytic and histiocytic type. In two instances abnormal cells, mainly histiocytes, were confined to the periphery of the malpighian bodies and to the peri-arteriolar areas (Fig. 3). In the other spleen atypical cells were present throughout the splenic lymphoid tissue (Fig. 4).

The 5 spleens with single or only a few macroscopic foci of tumour tissue were from patients with either lymphocytic predominant (1 case), nodular sclerotic (2 cases) or mixed type disease (2 cases) in the original lymph node. Only in one of the latter patients was the histology of the small focus in the spleen the same as in the node, 2 were lymphocytic depleted although the nodes were lymphocytic predominant and nodular sclerotic while 2 were lymphocytic predominant with the original node being nodular sclerotic and mixed.

Study of all the invaded spleens confirmed that the earliest invasion was in the peri-arteriolar area (Fig. 5) and the malpighian body, either confined to the periphery or throughout. Invaded malpighian bodies were in almost every instance enlarged and in these early stages did not show the banded fibrosis of nodular sclerosis, although diffuse fibrosis with lymphocytic depletion was occasionally seen. With progression of the disease the malpighian bodies gradually enlarged and coalesced to form larger foci. It was usually at this stage that bands of fibrous tissue were seen in the nodular sclerotic type.

The histological type of Hodgkin's disease in any one spleen may vary and for example one case showed classical nodular sclerotic disease in one area (Fig. 6), but lymphocytic predominant disease confined to the peri-arteriolar zone and malpighian bodies in a second area (Fig. 7) and collections of " Hodgkin's granulomata " in a third area (Fig. 8). In patients with nodular sclerotic Hodgkin's there appeared to be a slightly greater degree of diffuse fibrosis in the spleen compared with the original lymph node biopsy.

*Abdominal lymph nodes*

A total of 20 of the 44 patients with pre-treatment laparotomies had abdominal lymph node biopsies. Fourteen were invaded by Hodgkin's tissue the size of these nodes varying and on occasion being under 1 cm. In 11 of these patients the spleen was also involved by Hodgkin's tissue. The lymph node sites involved were in 5 of 6 instances splenic, 8 of 12 para-aortic and a single coeliac node biopsy, while a solitary mesenteric node was uninvolved. In one patient the abdominal node was the original diagnostic biopsy but in the other 13 cases the abdominal node histology was compared with the original extra abdominal lymph node and in all but three instances was found to be of the same histological type. Two of these exceptions showed nodular sclerosis originally but a mixed cellularity in the abdominal lymph nodes and no fibrosis although one of these nodes contained lacunar cells. The third patient with mixed type disease in the original lymph node showed a lymphocytic depleted histology in the abdominal node. Study of the lymph nodes of patients with nodular sclerosis showed that in some instances the abdominal lymph nodes had minimal fibrous banding although the original node had typical dense fibrosis. Others were more diffusely fibrotic in type. In two biopsies small localised foci of Hodgkin's tissue were present in the para-follicular area of the lymph node (Fig. 9).

*Liver biopsies*

Forty-eight of the 50 patients in this study had wedge liver biopsies. In 5 biopsies small foci of Hodgkin's tissue were present, as illustrated in Fig. 10. The livers of 2 other patients were seen at operation to contain nodules of tumour although the biopsies were uninvolved. In addition to those cases with definite foci of abnormal cells containing diagnostic Reed-Sternberg cells, 2 liver biopsies showed a moderate portal mononuclear cell infiltrate, sometimes with occasional eosinophils, which extended out into surrounding parenchyma (Fig. 11). In the absence of diagnostic cells these cellular infiltrates were difficult to interpret. Two patients with invaded spleens and 2 with uninvolved spleens showed a moderate chronic inflammatory cell infiltrate in the portal tracts while in 24 biopsies, 14 of which were associated with invaded spleens, the cellular infiltrate was only mild. In 5 invaded and 8 uninvolved spleens no inflammatory cells were present in the portal tract.

### DISCUSSION

Laparotomy performed as a diagnostic procedure in Hodgkin's disease has revealed that clinical assessment of splenic, liver and abdominal lymph node involvement may be inaccurate (Glatstein *et al.*, 1969, 1970; Jelliffe *et al.*, 1970). In any individual the enlargement of the spleen does not necessarily indicate invasion nor does an impalpable spleen exclude involvement. Even on pathological examination small foci of Hodgkin's tissue may be missed easily unless the spleen is sliced very carefully and on occasion invasion will only be revealed on microscopic examination. Multiple sections of the liver and abdominal lymph node biopsies may be needed before Hodgkin's tissue is detected.

In this study no correlation was found between the histological type of Hodgkin's disease in the original lymph node and the likelihood of invasion of the spleen. A higher percentage of patients with the histological types considered to have a better prognosis, *i.e.* lymphocytic predominance and nodular sclerosis, had invasion of the spleen compared with the mixed cellularity group. The number of patients studied is small but this could suggest that although these patients have widespread disease at the onset they may be able to resist it more effectively than patients with mixed cellularity and lymphocytic depleted disease. It is not possible to assess at this time whether the removal of spleens with only small foci is an effective part of treatment. The extent of splenic involvement in the 6 patients with delayed laparotomies varied with some showing massive involvement while 2 showed only occasional nodules even after 3 and 13 years respectively since the original lymph node biopsy.

Application of the Rye histological classification of Hodgkin's disease to the spleen proved relatively simple. Almost half the invaded spleens removed before treatment showed the same histological type in the spleen as in the lymph node, the greatest percentage with similar features being in the lymphocytic predominant group. No spleen showed characteristics of nodular sclerosis without a similar histology in the original lymph node. A few cases showed a more lymphocytic predominant picture in the spleen compared with the lymph node but this may reflect an earlier stage of involvement of the former organ. The significance of a " worse " type of histology in the spleen, *i.e.* lymphocytic depleted, compared with the lymph node is difficult to assess and may reflect a tendency for the splenic lesions to become more diffusely fibrotic. However it is possible that these patients are those in which the Hodgkin's disease might progress more rapidly.

The majority of spleens with early involvement showed a lymphocytic predominant type of disease although a case each of mixed cellularity and lymphocytic depleted types were seen. In some spleens with early involvement atypical cells appeared initially confined to the peri-arteriolar lymphoid tissue and the periphery of the malpighian bodies. This suggests that these areas are the sites of earliest invasion and that diffuse infiltration of the lymphoid tissue occurs subsequently. Following this the malpighian bodies gradually enlarge and coalesce to form larger nodules. The peri-arteriolar area is generally accepted as the site of thymic dependent lymphocytes in the spleen and it is interesting that the present authors have also noticed that the earliest involvement of lymph nodes by Hodgkin's disease may be confined to the parafollicular or thymic dependent area. These histological appearances in the lymph nodes and spleens suggest the possibility that the thymus may have a role in Hodgkin's disease.

Nodular sclerotic Hodgkin's disease with dense fibrous bands and characteristic lacunar cells was seen in the spleens of 5 of 11 patients with nodular sclerosis in the original lymph node biopsy. The cellular component varied in a similar manner to lymph nodes, having either a lymphocytic predominant mixed cellularity or lymphocytic depleted type picture although there was a tendency for this cellular element to be slightly more diffusely fibrotic than the original lymph node biopsy. A fairly dense rim of iron pigment was sometimes present in the bands of fibrous tissue. The features of nodular sclerosis were not always present throughout the spleen and areas of early involvement of the lymphoid tissue by a lymphocytic and histiocytic predominant type of disease with no fibrosis were seen, although sometimes lacunar cells were present. It is interesting that in 2 of the patients in whom laparotomy was delayed the spleen was involved by nodular sclerotic type disease with a lymphocytic predominant cellular component although the original diagnosis had been made 3 and 13 years previously. Both these spleens were of normal weight with only scattered nodules of tumour up to 1 cm. Particularly in the longer surviving case extension of disease to or within the spleen must have occurred slowly.

A mixed cellularity type Hodgkin's disease was present in only 2 spleens one containing 5 foci and the other numerous nodules of tumour. Similarly lymphocytic depleted histology was seen in spleens with both extensive invasion and with only a few small foci present.

*Abdominal lymph nodes*

The abdominal lymph nodes biopsied demonstrated that even the smallest may show microscopic evidence of invasion by Hodgkin's disease and that consequently any accessible lymph node whatever size should be biopsied. In 10 of 13 patients the same histological type of Hodgkin's disease was seen in the abdominal lymph node as in the original lymph node. The abdominal lymph nodes of 10 appeared to have earlier and less extensive involvement than the original lymph node. This may account for 2 nodular sclerotic cases not showing bands of fibrous tissue in the abdominal lymph nodes.

*Liver*

The decision as to whether the liver is involved is of considerable importance to the patient as it may affect the type of treatment given but histological assessment has proved difficult in this study. In all but one of the definitely invaded biopsies foci of abnormal cells were small and careful search was needed to detect Reed-Sternberg cells. In 26% of biopsies no portal tract infiltrate was present, but in nearly half there was a mild chronic inflammatory cell infiltrate. In a further 4 biopsies the infiltrate was classified as moderate, but was confined to the portal tracts. In 2 instances the cellular infiltrate also extended out amongst the liver parenchymal cells and contained some large abnormal histiocytes, but despite careful search no Reed-Sternberg cells were found. The present authors are concerned that these mixed cellular infiltrates may indicate early infiltration by Hodgkin's disease.

It was surprising that a high percentage of patients with lymphocytic predominant disease in the original lymph node had invaded livers. A greater percentage of cases with delayed laparotomies had invaded livers compared with the pre-treatment laparotomies as might be expected.

The authors wish to thank the many clinicians and surgeons who referred patients for investigation and treatment. We are very grateful to Miss Jenny Abrahams, Miss Eileen Green and Miss Linda Dean for secretarial assistance. Financial support for this work and for the Clinical Cancertherapy Cooperative Group has been generously provided by Eli Lilly & Co., Roche Products Ltd., the J. S. Frazer Trust Fund, the Peggy Russell Memorial Fund, the New Court Charitable Trust, the Aitchison Charitable Trust, the Cancer Research Campaign and many individuals who have a personal interest in this research programme.

### REFERENCES

Glatstein, E., Guernsey, J. M., Rosenberg, S. A. and Kaplan, H. S.—(1969) *Cancer, N.Y.*, **24**, 709.

Glatstein, E., Trueblood, H. W., Enright, L. P., Rosenberg, S. A. and Kaplan, H. S.—(1970) *Radiology*, **97**, 425.

Jelliffe, A. M., Millett, Yvonne, L., Marston, J. A. P., Bennett, M. H., Farrer-Brown, G., Kendall, B. and Keeling, D. H.—(1970) *Clin. Radiol.*, **21**, 439.

Lukes, R. J. and Butler, J. J.—(1966) *Cancer Res.*, **26**, 1063.

Lukes, R. F., Craven, L. F., Hall, T. C., Rappaport, H. and Rubin, P.—(1966) *Cancer Res.*, **26**, 1311.


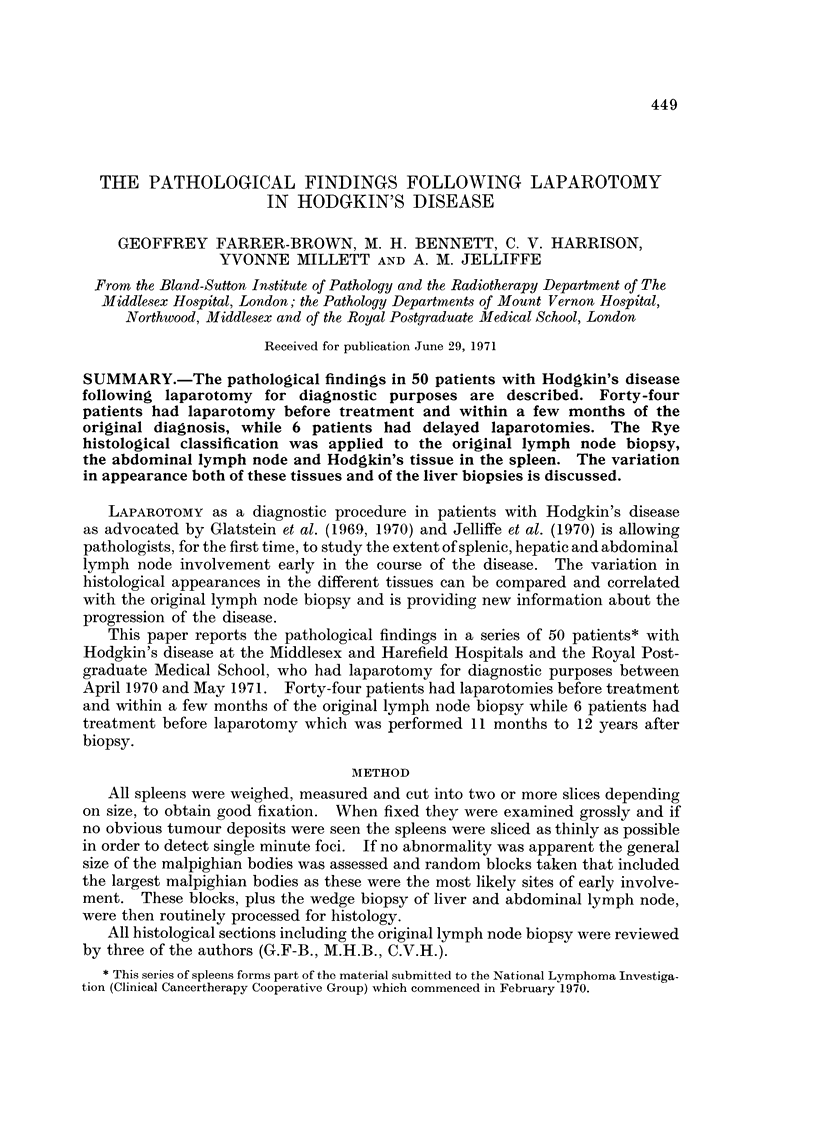

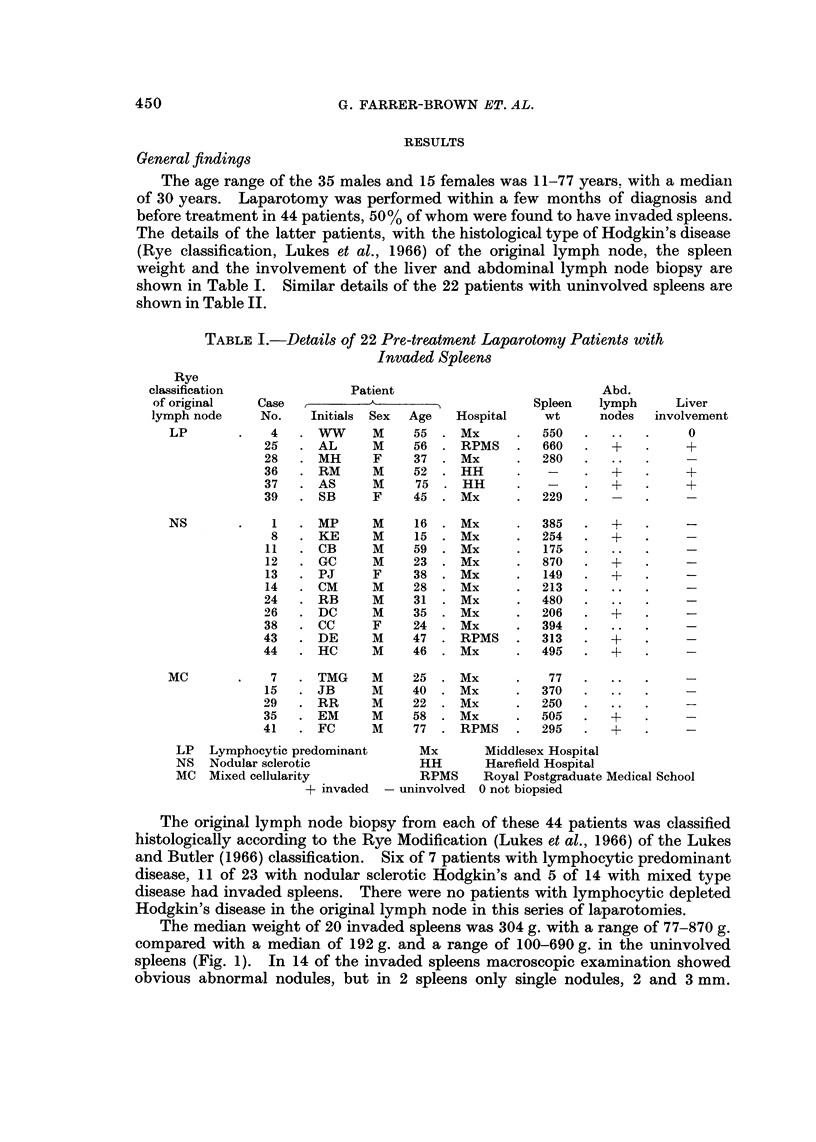

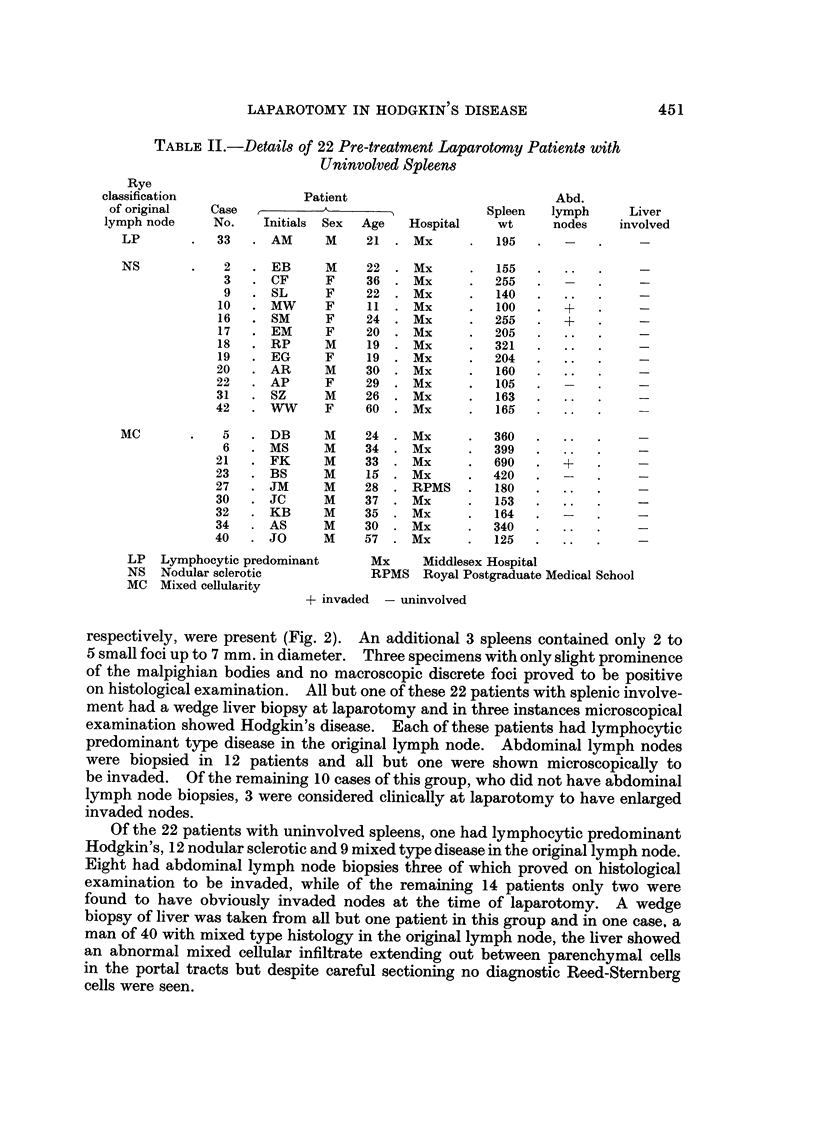

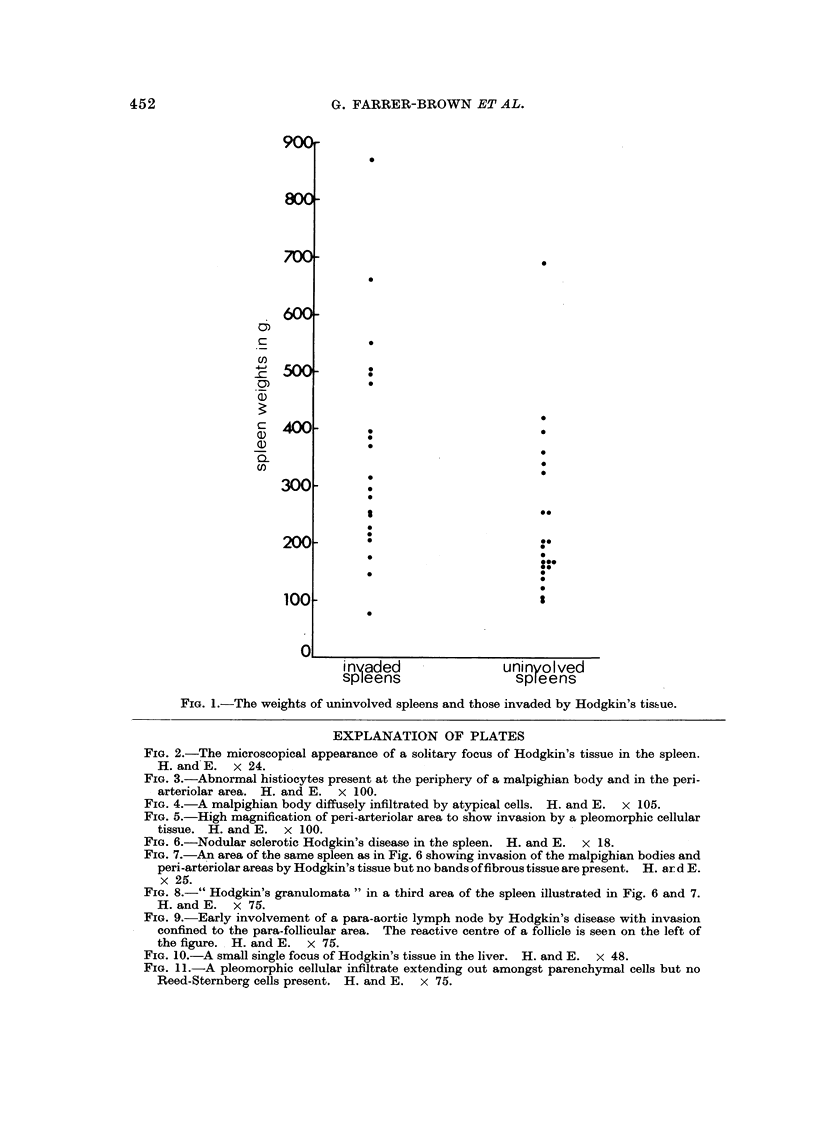

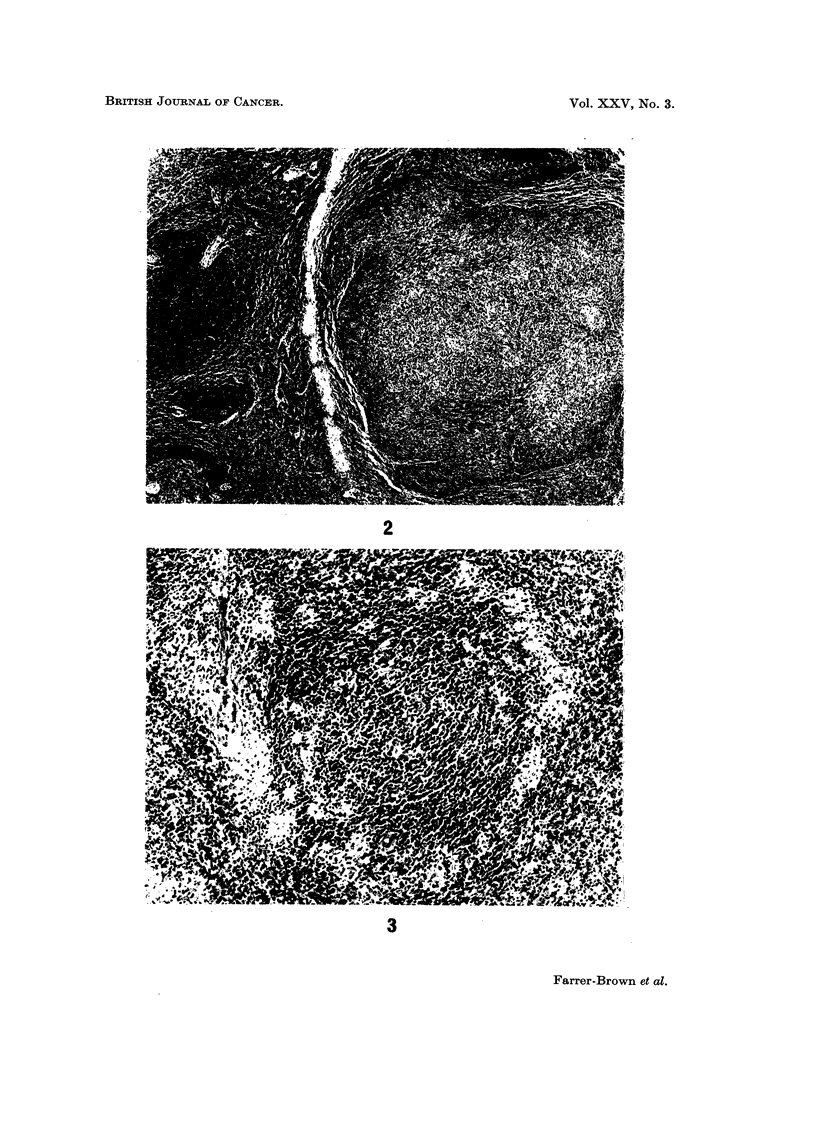

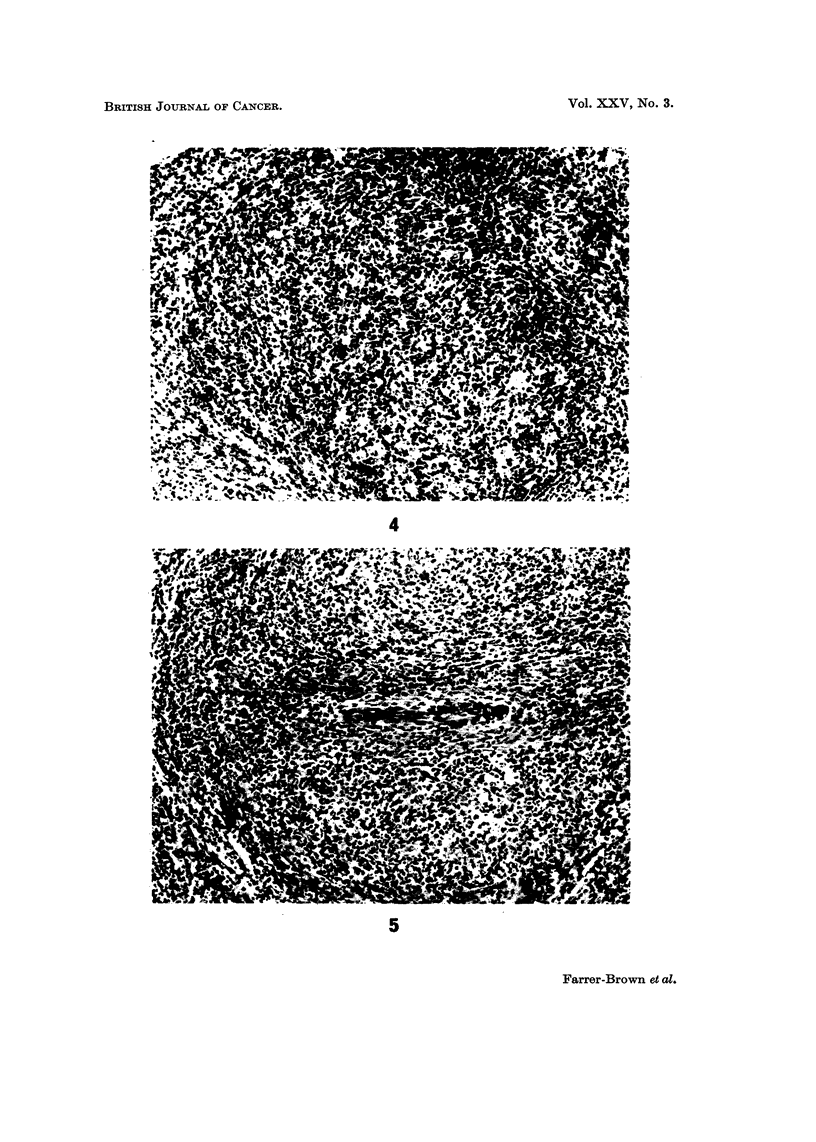

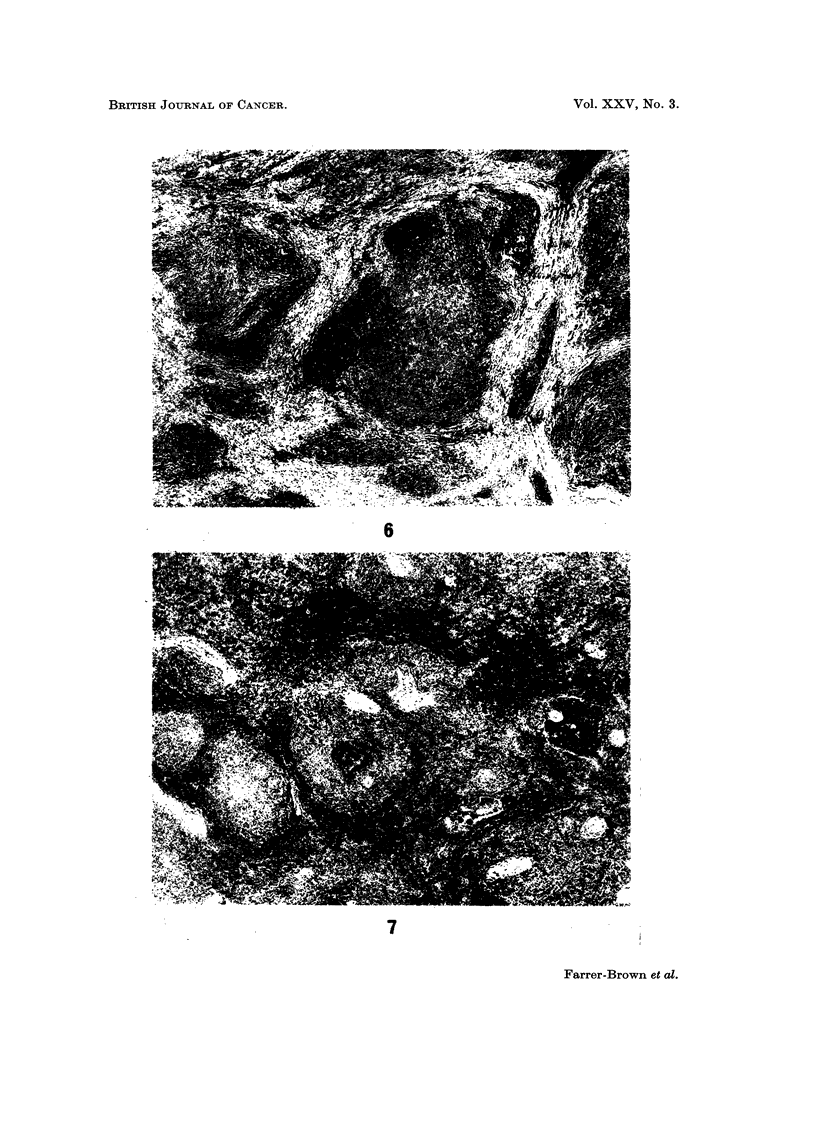

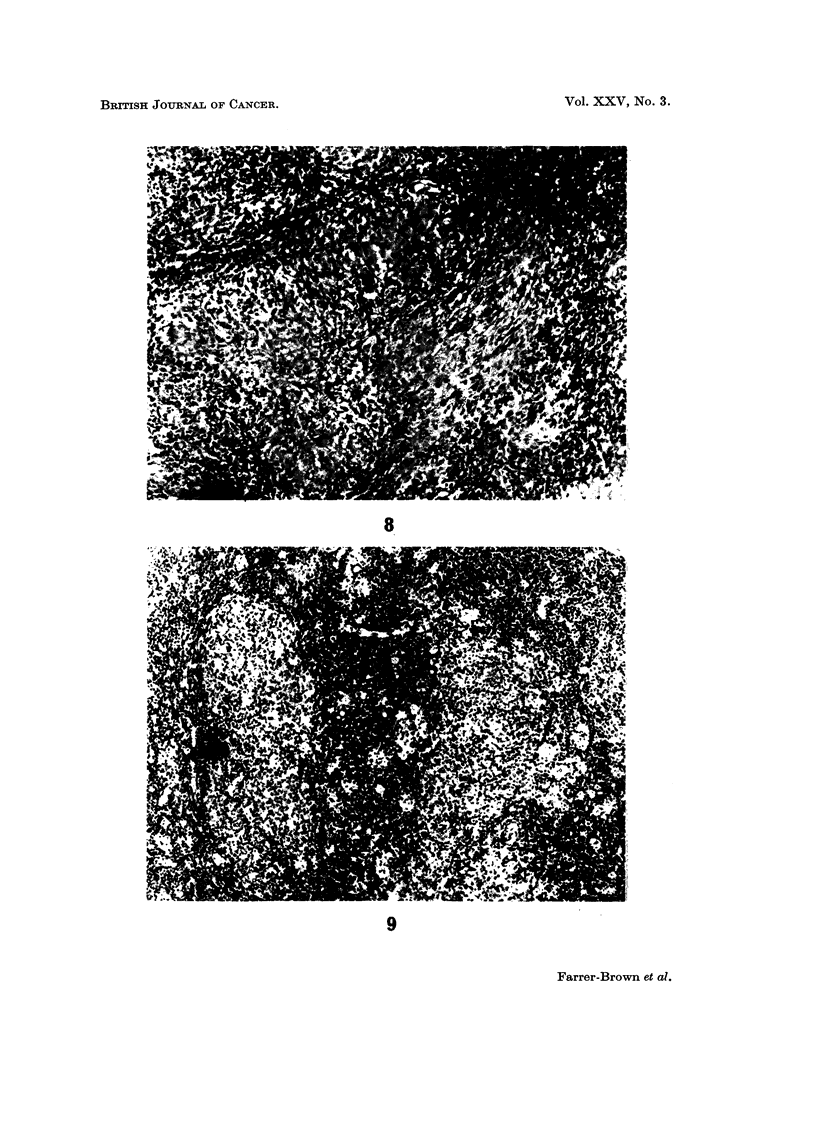

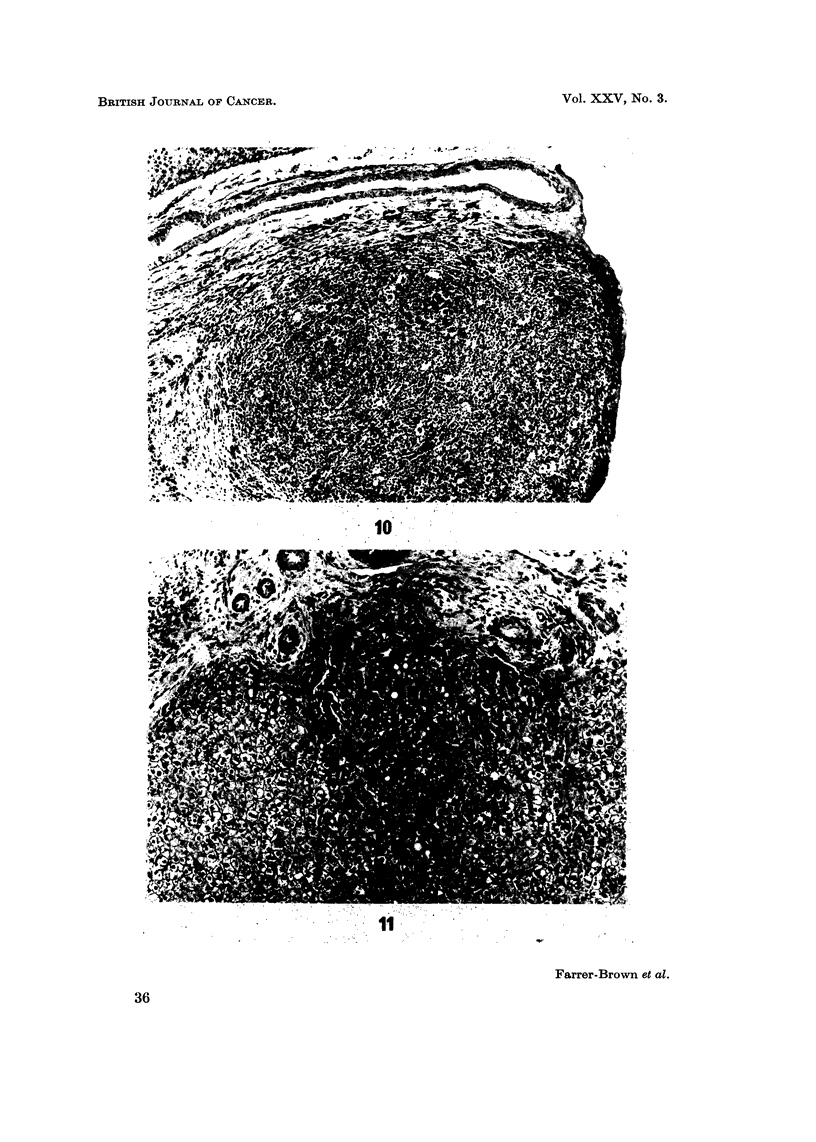

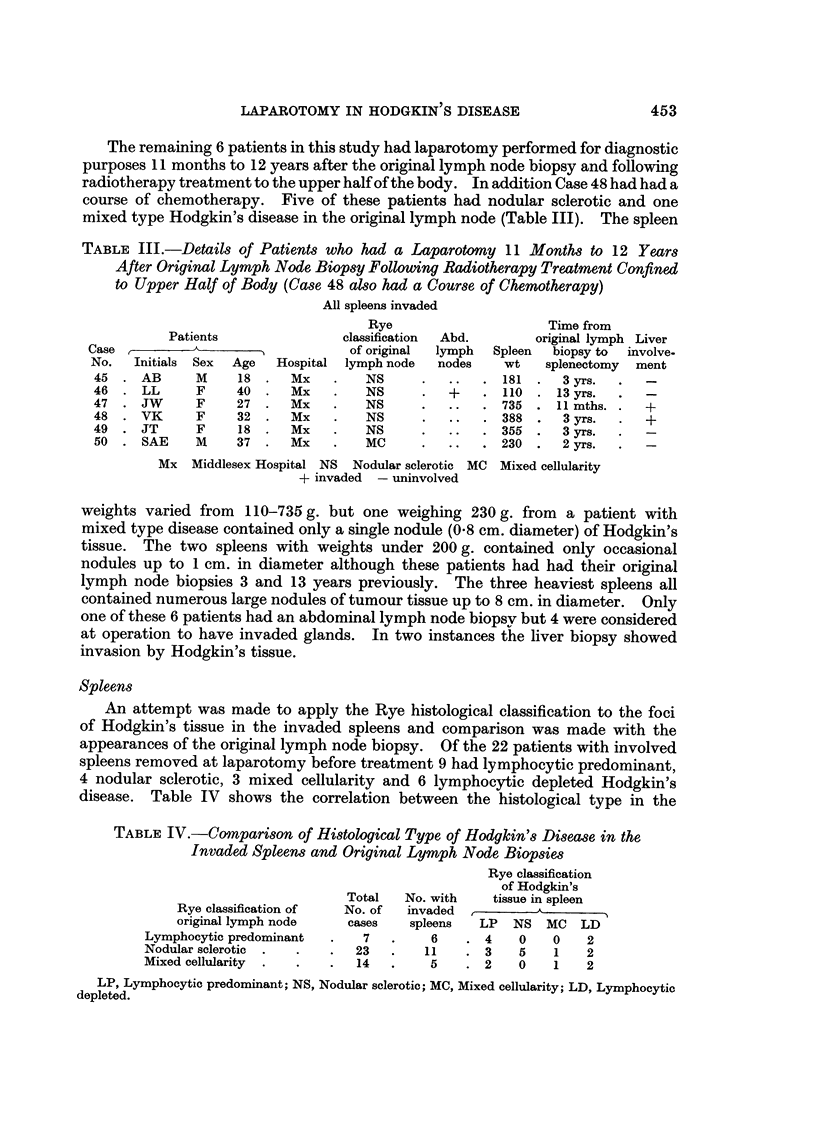

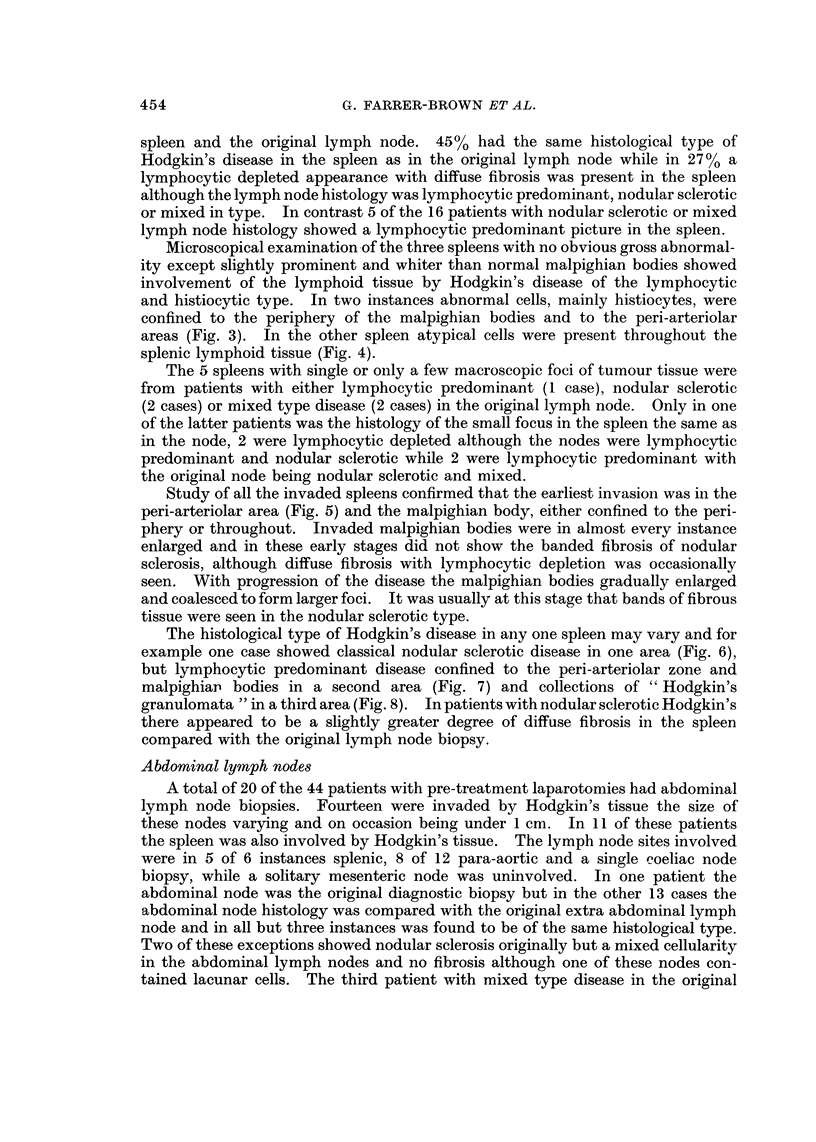

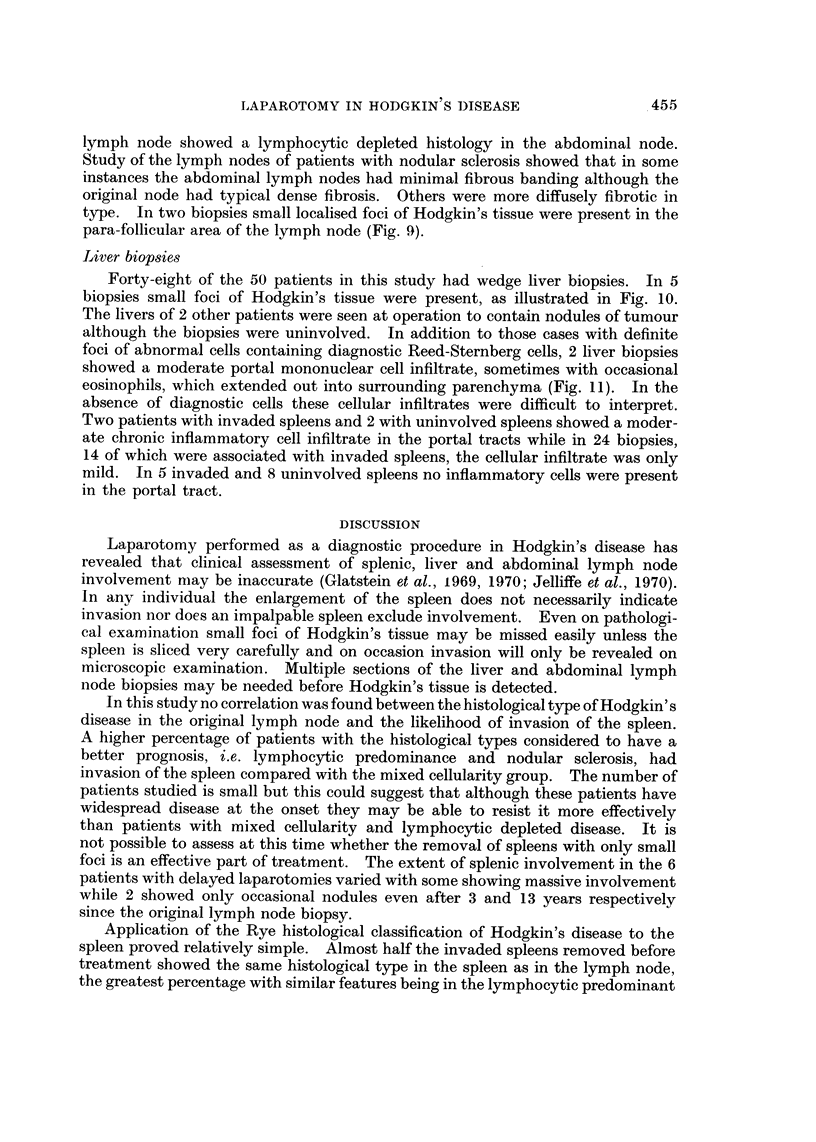

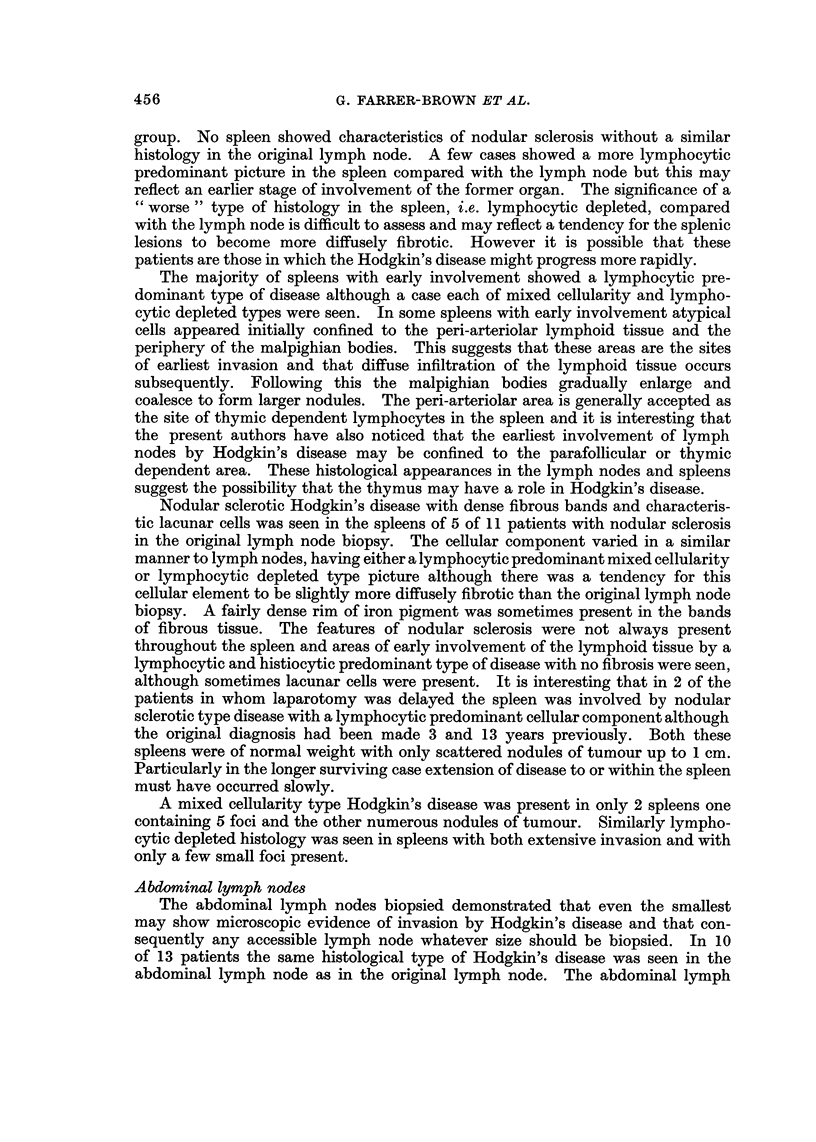

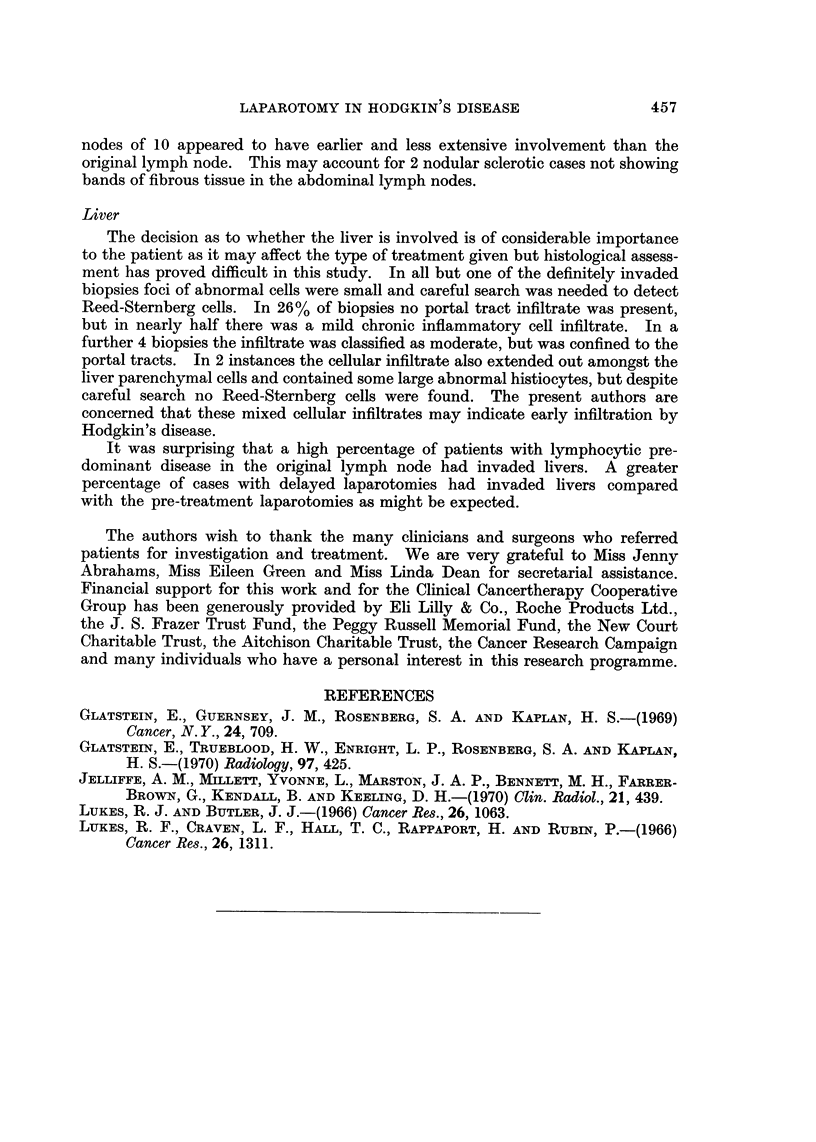


## References

[OCR_00845] Glatstein E., Guernsey J. M., Rosenberg S. A., Kaplan H. S. (1969). The value of laparotomy and splenectomy in the staging of Hodgkin's disease.. Cancer.

[OCR_00847] Glatstein E., Trueblood H. W., Enright L. P., Rosenberg S. A., Kaplan H. S. (1970). Surgical staging of abdominal involvement in unselected patients with Hodgkin's disease.. Radiology.

[OCR_00851] Jelliffe A. M., Millett L., Marston J. A., Bennett M. H., Farrer-Brown G., Kendall B., Keeling D. H. (1970). Laparotomy and splenectomy as routine investigations in the staging of Hodgkin's disease before treatment.. Clin Radiol.

[OCR_00854] Lukes R. J., Butler J. J. (1966). The pathology and nomenclature of Hodgkin's disease.. Cancer Res.

